# Effects of Global Postural Re-Education Versus Specific Therapeutic Exercises on Pain, Head Posture, and Pain-Related Psychosocial Factors in Women with Chronic Nonspecific Neck Pain: A Randomized Clinical Trial

**DOI:** 10.3390/jcm14051581

**Published:** 2025-02-26

**Authors:** Tânia Fernandes, Carolina Vila-Chã, Luis Polo-Ferrero, Javier Martín-Vallejo, Ana Silvia Puente-González, Roberto Méndez-Sánchez

**Affiliations:** 1 Centro EMA, 6300-406 Guarda, Portugal; taniafernandes_ft@hotmail.com; 2 School of Sport, Well-Being and Biomedical Systems, Polytechnic Institute of Cávado and Ave (IPCA), 4810-453 Guimarães, Portugal; cvila-cha@ipca.pt; 3 Sport Physical Activity and Health Research & Innovation Center (Sprint), 2040-413 Rio Maior, Portugal; 4 Institute of Biomedical Research of Salamanca (IBSAL), 37007 Salamanca, Spain; pfluis@usal.es (L.P.-F.); jmv@usal.es (J.M.-V.); ro_mendez@usal.es (R.M.-S.); 5 Department of Nursing and Physiotherapy, University of Salamanca, 37007 Salamanca, Spain; 6 Department of Statistic, University of Salamanca, 37007 Salamanca, Spain

**Keywords:** neck pain, global postural re-education, therapeutic exercise, chronic pain, mechanosensitivity, kinesiophobia, pain catastrophizing, disability

## Abstract

**Background**: The aim of this trial is to compare the effects of two types of exercises, Global Postural Re-education versus specific therapeutic exercises on pain perception, pain threshold to pressure, psychosocial factors associated with pain, and craniocervical posture in women with chronic nonspecific neck pain. **Methods**: This study is a randomized, parallel-group, single-blind clinical trial. Fifty-two women with chronic nonspecific neck pain (two excluded) were recruited and randomly assigned to (n = 25) Global Postural Re-education and (n = 25) specific therapeutic exercises. Interventions were carried out for 4 weeks, two sessions per week, and were combined with a daily home exercise program. Numerical Pain Rating Scale, mechanosensitivity to pressure, kinesiophobia, pain catastrophizing, and craniocervical angle were assessed in two pre-intervention assessments, one week apart, and at 2 and 4 weeks after the start of the intervention. **Results**: Global Postural Re-education was as effective as specific therapeutic exercises, showing improvements in all variables assessed with significant intra-group differences over time and high effect sizes (ŋp^2^ > 0.157 for all variables). **Conclusions**: Global Postural Re-education is as effective as a specific therapeutic exercise program in reducing subjective pain perception, local and remote mechanosensitivity, and short-term pain-related psychosocial factors in women with chronic nonspecific neck pain.

## 1. Introduction

Neck pain is one of the most common musculoskeletal conditions [1,2,3], it is estimated that, according to epidemiological data, up to 70% of the population will suffer from it at some point in their lives [4], and it is more prevalent in women [4,5].

Neck pain is a prevalent musculoskeletal condition, affecting up to 70% of the population at some point in life, with a higher prevalence in women [1,2,3,4,5]. Chronic nonspecific neck pain (CNSNP) is defined as pain persisting for over 12 weeks without an identifiable pathology and is associated with disability, muscle dysfunction, reduced cervical range of motion (CROM), and increased pain sensitivity [6]. It ranks as the fourth-leading cause of disability globally [7], with 50% of affected individuals continuing to experience symptoms after one year [8]. Additionally, CNSNP imposes a significant burden on public health systems [9], making it a sociohealth problem [6].

The persistence of CNSNP is multifactorial, involving sensory–motor alterations and psychosocial components such as anxiety, depression, and fear of movement [6,10,11,12,13,14,15,16,17]. Its etiology is not fully understood, though dysfunction and/or sensitization of cervical structures may play a role [18], with increased mechanosensitivity of the cervical region and median nerve being a common finding in affected individuals [19]. Neuromuscular alterations, particularly myofascial trigger points (MTrPs) in the upper trapezius [20,21,22,23,24,25], along with abnormal contraction patterns in cervical flexor muscles [26,27,28,29,30] and reduced strength/endurance of deep cervical flexors (DCF) [31,32,33,34], contribute to postural imbalances. Proprioceptive and kinesthetic deficits [13,35,36,37,38] further exacerbate postural dysfunction, notably forward head posture, which is characterized by a reduced craniocervical angle (CCA) and is strongly linked to neck pain [24,39,40].

Postural alterations may increase tissue mechanosensitivity [41,42] and are also associated with reduced cervical mobility [3,13,43,44] and sensitization of the peripheral and central nervous systems [3,45,46,47,48]. Central sensitization refers to an amplified response of the central nervous system to stimuli, leading to increased pain sensitivity and altered pain processing, even in the absence of ongoing tissue damage [49]. Given this, evaluating central sensitization mechanisms, particularly through assessments of widespread pressure pain hyperalgesia, is crucial in CNSNP [50,51,52].

Psychosocial factors play a crucial role in musculoskeletal chronic pain, with central sensitization being a key mechanism [50,53]. Patients with CNSNP often exhibit altered pain cognitions, including kinesiophobia and catastrophizing, which are linked to increased pain intensity and disability [50,54,55].

Several nonpharmacological treatments, including manual therapy and therapeutic exercise, have demonstrated benefits in CNSNP management [56,57,58,59]. Additional interventions, such as pain neuroscience education, dry needling, shock waves, and multimodal biopsychosocial approaches, have also been recommended to reduce disability [60,61,62,63,64]. While specific therapeutic exercise (STE) has shown positive effects on pain, disability, mobility, and muscle performance [56,57,59,65], the most effective type of exercise for neck pain remains unclear [66,67]. Further research is needed to compare different exercise modalities, particularly considering central sensitization mechanisms.

Global Postural Re-education (GPR) is a global exercise approach based on the coordination of muscle chains [68,69,70,71,72,73], aiming to restore muscular balance through prolonged postures, antagonist muscle contractions, breathing control, and sensory–motor integration, with manual guidance from a physiotherapist [69,73,74,75,76,77]. GPR is widely applied in clinical practice [69,72,78,79,80] and has demonstrated efficacy in musculoskeletal disorders [69,70,72,78,81,82,83,84,85], particularly in reducing pain and disability in CNSNP [70,86,87]. However, evidence remains limited due to study heterogeneity and variable quality [72,78,80]. Its effects on psychosocial factors, such as kinesiophobia, are inconsistent [70,86], and no studies have assessed its impact on generalized pressure pain hyperalgesia, although it may enhance cortical inhibition in related peripheral muscles [83].

No prior studies have compared GPR with other exercise modalities, such as strengthening and motor control exercises, which have already shown benefits in CNSNP. Investigating whether active postural exercises could be a viable alternative for neck pain management is essential [87]. This study aims to compare the effects of GPR and a specific therapeutic exercise program on neck pain, hypersensitivity to pressure, kinesiophobia, catastrophizing, and disability in individuals with CNSNP, hypothesizing that GPR may yield superior outcomes.

## 2. Materials and Methods

### 2.1. Study Design

This investigation was designed as a randomized, parallel-group, single-blinded controlled clinical trial. The study protocol adhered to the SPIRIT 2013 guidelines [88], ensuring methodological rigor. Ethical approval was granted by the Ethics Committee of the University of Salamanca (protocol ID: 458-2019 on 21 November 2019), following the principles of informed consent. The trial was prospectively registered on ClinicalTrials.gov (NCT04402463, prospectively registered (22 May 2020)) and conducted in strict compliance with the CONSORT 2010 guidelines (Consolidated Standards of Reporting Trials) [89], as well as the ethical principles outlined in the Declaration of Helsinki.

### 2.2. Participants

A total of 52 women with CNSNP were recruited through an online Google form disseminated via social networks within Guarda County, Portugal, and via email invitations sent to staff at the Polytechnic Institute of Guarda. Two assessor physiotherapists reviewed the responses from individuals expressing interest in participating. During an initial visit, potential participants were thoroughly informed about the study’s objectives and procedures. After voluntarily providing written informed consent, eligibility was confirmed based on the inclusion criteria (women aged 18–65 years with CNSNP persisting for at least 12 weeks) and exclusion criteria (diagnosed specific neck pain, history of spinal surgery, central or peripheral neurological disorders or symptoms, physiotherapy for neck pain within the past three months, or ongoing pharmacological treatment). Participants were also excluded if they initiated any additional treatments during the study. Ultimately, two individuals did not meet the study protocol requirements and were excluded, resulting in a final sample of 50 participants (50.82 ± 8.77 years) who underwent baseline assessments of dependent and independent variables under the supervision of the assessor physiotherapists.

The required sample size was determined based on the anticipated changes in the primary outcome variables, neck pain intensity and neck disability (NPRS and NDI-PT), from baseline to the final assessment. A priori sample size calculation was performed to ensure adequate statistical power for detecting clinically meaningful differences in these outcomes. For repeated measures analysis, a minimum of 26 participants per group was estimated, assuming an alpha risk of 0.05 and a beta risk of 0.2 in a two-tailed test, corresponding to a statistical power of 80%.

The effect size assumptions were derived from previous studies analyzing similar interventions for chronic nonspecific neck pain, which reported expected changes of approximately 2 points in NPRS (SD = 1.75) and 7 points in NDI (SD = 6) as clinically relevant thresholds. These values were selected based on their representation of minimal clinically important differences (MCID) in chronic neck pain management. This sample size was designed to detect a statistically significant difference of at least 2 units in NPRS or 7 units in NDI, ensuring the ability to identify moderate-to-large effect sizes between groups (Cohen’s d = 0.8).

Additionally, a 15% potential dropout rate was accounted for in the calculation. The final estimated sample size was considered sufficient to ensure robust statistical analysis and minimize the risk of type II errors. The sample size estimation was conducted using the “PASS 15” software (NCSS statistical software) and the R Project version 4.3.0 (The R Project for Statistical Computing, Vienna, Austria). for Statistical Computing.

### 2.3. Randomization and Blinding

Following the second pre-intervention assessment and prior to initiating the interventions, an independent evaluator conducted the randomization process to allocate participants into the GPR and STE groups. Randomization was performed using a computerized system (randomized.org), ensuring allocation concealment through sequentially numbered, opaque, sealed envelopes.

Blinding procedures were implemented to minimize bias. The assessor responsible for measuring all outcome variables remained blinded to group allocation during each evaluation. However, due to the nature of the interventions, participants were not blinded; they were informed that they would receive an effective exercise treatment but were unaware of the specific type of exercise assigned to their group.

### 2.4. Evaluations

**Figure 1:**
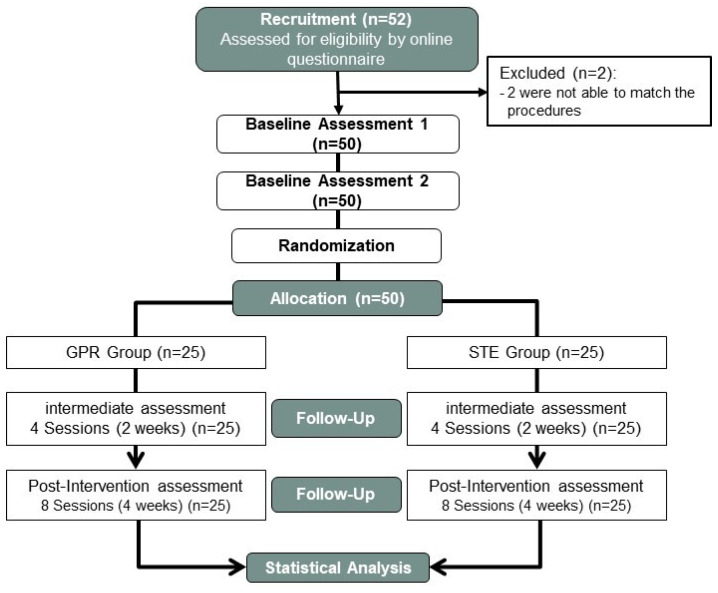
CONSORT flow diagram of participants in the study.

Baseline and follow-up assessments were conducted at four distinct time points during the study at the Polytechnic Institute of Guarda, as illustrated in Figure 1. To account for day-to-day variability, two pre-intervention assessments were performed one week apart before the intervention commenced. Following the second pre-intervention evaluation, participants initiated a four-week intervention program consisting of two sessions per week.

An intermediate assessment was conducted after the fourth treatment session to monitor progress, while the final evaluation took place at the conclusion of the eighth session. The intermediate assessment was limited to pain-related variables, specifically neck pain intensity and pressure pain threshold (PPT) at the designated measurement points.

### 2.5. Outcome Variables

Personal and sociodemographic variables were recorded in the first pre-intervention assessment, along with the primary and secondary outcome variables, which were evaluated under standardized conditions in all follow-ups.

The primary outcome was neck pain intensity (Numerical Pain Rating Scale, NPRS). This measure was selected for its extensive validation, reliability, and sensitivity to clinically meaningful changes in chronic pain [90,91], specifically in CNSNP [92]. The secondary outcomes included cervical range of motion (CROM), pressure pain threshold (PPT), and psychosocial factors (kinesiophobia and catastrophization). CROM was assessed due to its relevance in evaluating functional limitations in CNSNP. PPT was measured at the cervical spine, upper trapezius, and tibialis anterior to assess peripheral and central sensitization, as it is an objective and validated marker of mechanosensitivity. Kinesiophobia and catastrophization were included for their strong influence on chronic pain persistence and recovery, using validated scales commonly applied in musculoskeletal research.

#### 2.5.1. Primary Outcome Variables

Neck pain intensity: It was measured using the NPRS, as it is demonstrated to be a valid and reliable tool [90,91,92], with an 11-point scale ranging from 0 (“no pain”) to 10 (“worst pain imaginable”), considering the pain score over the week prior to the evaluation.

#### 2.5.2. Secondary Outcome Variables

1. Mechanosensitivity: It was assessed using the PPT, measured with a digital algometer (Force Ten™-Model FDX; Wagner, Greenwich, CT, USA) featuring a 1 cm^2^ round tip surface. This device [93] was used to quantify PPT (kgf) at specific bilateral assessment sites: the paravertebral muscles adjacent to the second (C2) and sixth (C6) cervical vertebrae (prone position) and the upper trapezius muscle (sitting position) to evaluate peripheral sensitization, as well as the tibialis anterior muscle (supine position) to assess central sensitization [94,95,96,97]. The evaluator progressively increased pressure at each site until the participant reported the onset of pain [98]. PPT measurement via algometry has demonstrated excellent test–retest reliability (ICC = 0.91, 95% CI 0.82–0.97) [99].
2. Head Posture: To assess the head position, the craniocervical angle (CCA) was measured using a universal goniometer device (Sammons Preston-Rolyan, Bolingbrook, IL, USA). This angle is obtained from a line connecting C7 with the tragus and the horizontal line in the standing position. The measurement was carried out 3 times considering the mean value. The CCA is a widely used surface-based measurement for assessing head posture, particularly in individuals with cervical dysfunction. Previous studies have demonstrated its acceptable (ICC ≥ 0.71) to good (ICC ≥ 0.85) intra-rater reliability [98,100]. Additionally, research comparing CCA with radiographic indices has reported moderate to high correlations, supporting its validity as a clinical tool for evaluating postural alignment [101].
3. Pain-related psychosocial factors: To evaluate attitudes and thoughts related to pain, we assessed the influence of kinesiophobia and pain catastrophizing toward CNSNP:
  1. Kinesiophobia: This was measured using the Portuguese version of the 13-item Tampa Kinesiophobia Scale (TSK-13-PT) that assesses fear of movement and re-injury, with a very good consistency and test–retest reliability [102]. It consists of 13 items and each item is scored from 1 to 4 points. A higher score indicates higher levels of kinesiophobia, and it is classified into four ranges of intensity: ’subclinical’ (13–22); ’mild’ (23–32); ’moderate’ (33–42); and ’severe’ (43–52) [103].
  2. Catastrophizing: To measure pain catastrophizing, as a tendency to magnify the threat value of a painful stimulus and to feel helpless in the presence of pain, the Portuguese version of the pain catastrophizing scale (PCS-PT) was used, also with very good internal consistency and test–retest reliability [104,105]. This scale is composed of 13 items, each scored from 0 to 4 points (none to all the time). Participants have to describe the frequency with which they experience different thoughts and feelings associated with pain that are grouped into 3 subscales: rumination (4 items), magnification (3 items), and helplessness (6 items) [106,107].

### 2.6. Interventions

Participants in each study group underwent distinct intervention protocols (GPR and STE), both administered by an experienced physiotherapist specializing in musculoskeletal disorders.

These interventions involved two different exercise modalities: GPR, which focused on global static postural exercises, and STE, which targeted motor control specific to the cervical region. A detailed description of these protocols can be found in the corresponding study protocol publication [108]. Comparing them allows for assessing whether a global approach like GPR is as effective as the specific one, expanding exercise-based therapeutic options.

Both interventions commenced following the second pre-intervention assessment and were carried out over a four-week period. Each intervention consisted of a combination of supervised treatment sessions conducted by the physiotherapist and a complementary home exercise program. Participants attended eight supervised sessions, each lasting approximately 40 min, scheduled twice weekly with an interval of 72 to 96 h between sessions.

Additionally, all participants followed a home exercise regimen tailored to their assigned intervention, performed daily for four weeks. Compliance and any adverse events were documented in an exercise diary.

#### 2.6.1. Global Postural Re-Education (GPR)

Participants in this group engaged in global postural exercises comprising three distinct positions, following the protocol outlined by Lozano-Quijada et al. [109]. Initially, two postures were performed in a lying position without gravity load for 15–20 min, followed by an upright standing posture for 5 min, as already published in the study protocol [108].

The first posture targeted the anterior muscle chain, with shoulders abducted, forearms supinated, and the pelvis in a neutral position. The objective was to transition progressively from hip flexion and external rotation with knee flexion to full extension of the lower limbs. The second posture focused on the posterior muscle chain, maintaining a supine position with the lower limbs in 90° hip flexion, followed by a gradual extension of the knees assisted by a cord placed under the feet.

To conclude the session, participants held the upright standing posture for five minutes. Throughout all three postures, exercises were adapted according to the progression of load and difficulty. Participants performed isometric contractions (5–10 s), active postural adjustments, controlled breathing, stretching, manual traction administered by the physiotherapist, and sustained stretching, as described previously [73,109].

To adhere to the GPR principle of individualized treatment, adjustments were permitted regarding pause durations, manual contact variations, time allocated to each posture, and breathing techniques, ensuring an optimized intervention for each participant.

#### 2.6.2. Specific Therapeutic Exercise (STE)

Participants assigned to the STE group engaged in targeted motor control exercises for the cervical and axioscapular regions, structured into three progressive phases, as previously described [65,91,92]. Additionally, visual feedback was incorporated using a laser sensor (MotionGuidance^®,^ Denver, CO, USA) to enhance movement precision as described in the protocol study [108].

For cervical musculature training, exercises followed a gradual progression with low-load activation. Deep neck flexor exercises were performed in a supine position, utilizing an air-filled pressure sensor (Stabilizer™, Chattanooga Group Inc., Chattanooga, TN, USA) for biofeedback. Meanwhile, deep extensor exercises were executed in a prone position. Axioscapular exercises focused on strengthening the middle and lower trapezius muscle fibers, employing a three-stage progression that included scapular repositioning and sustained retraction and depression exercises.

Regarding the home exercise program, both groups received detailed guidance from the physiotherapist on a set of simple, equipment-free exercises lasting approximately 15 min per session. Participants in the GPR group performed the first supine posture from their supervised sessions, modifying shoulder abduction angles while maintaining deep and controlled breathing. In contrast, STE participants executed active neck movements and muscle stretches in a seated position, ensuring pain-free execution with controlled breathing. Each movement was performed for 10 repetitions, while stretches were maintained for 20 s.

### 2.7. Statistical Analysis

Descriptive statistics were presented separately for each group, with numerical variables expressed as means ± standard deviation and categorical variables summarized as frequencies and percentages. The primary change from baseline to four weeks post-treatment was assessed through an intention-to-treat approach for all outcome measures.

Baseline data were examined to identify potential between-group differences in categorical variables, including age, height, weight, and BMI, using independent *t*-tests. If significant differences were detected in any of these variables, they were incorporated as covariates in subsequent analyses. A two-way repeated measures ANOVA was employed to assess the effects of both interventions on the outcome variables. In this statistical model, the intervention type (GPR or STE) was treated as a categorical factor, while time was modeled as a repeated measure encompassing two pre-intervention assessments (baseline), an intermediate evaluation, and a final assessment. The interaction effect between intervention type and time on the dependent variables was tested, and when statistically significant, post hoc comparisons using the Sidak test were performed to determine specific differences within participants across time points. The Sidak post hoc test was applied to identify which mean pairs exhibited significant differences.

Pearson’s correlation coefficient (r) was used to examine relationships between numerical variables and to assess whether changes in these variables occurred concurrently at different study time points.

To quantify the magnitude of observed changes, effect sizes were calculated for both interventions. For the repeated-measures ANOVA, partial eta squared (ŋp^2^) was computed, with interpretation thresholds set at 0.01 (small), 0.06 (moderate), and >0.14 (large). Additionally, when applicable, Cohen’s d was used to assess effect sizes in pairwise comparisons.

A significance level of *p* ≤ 0.05 was established for all statistical tests, with results interpreted within a 95% confidence interval. Data analysis was conducted using IBM-SPSS statistical software (version 23.0).

## 3. Results

Fifty-two participants were recruited, and fifty were selected and randomized after signing the informed consent and considering the inclusion and exclusion criteria. Two subjects were excluded because they were unable to match the procedures. All participants who started the study completed the intervention, according to the group to which they were assigned, so adherence to treatment and assessments was 100%, and without any adverse events informed. The demographic and outcome data of the sample, by groups, in the first pre-intervention assessment, are shown in Table 1. In baseline data, in both pre-intervention assessments, there were only significant differences between groups in age (*p* = 0.015), which was considered in subsequent analyses as a covariate in the cases in which it was significant in the statistical model.

The study design included two pre-intervention assessments, one week apart, in order to be able to assess the effects over time that can be attributed to the start of the interventions in both groups. It was observed that, during the time between the two pre-intervention assessments, there were significant within-group differences in the pairwise comparisons of some variables (Table 2). However, it is clear from analyzing these changes and those after the second pre-intervention assessment that the interventions were the determining factors in the beneficial changes in the outcome variables.

In the main variable (NPRS), pain-related psychosocial factors and CCA, there were no significant within-group differences in the pairwise comparisons between the two pre-intervention assessments, except for pain-related catastrophizing, where there were differences in the GPR group (*p* = 0.002; CI: [2.068–10.812]), with a decrease in the score before the intervention (Table 2). In the outcome variables of pain pressure threshold, both in the trapezius and anterior tibialis muscles, as well as in the cervical vertebrae (C2 and C6), there was a small decrease in the threshold, even with significant differences in some of them (see Table 2). As will be seen below, this trend at the beginning of the intervention was clearly reversed.

### 3.1. Results of Neck Pain

**Figure 2:**
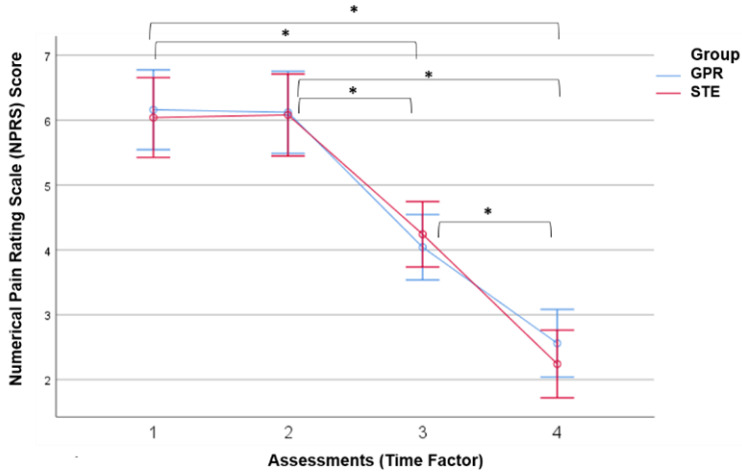
Monitoring of pain intensity across four assessment points in both intervention groups. GPR: Global Postural Re-education, STE: specific therapeutic exercise. * Statistically significant difference between assessments (pairwise comparisons by post hoc Sidak test (*p*-value < 0.001)).

The analysis of pain intensity (NPRS), by two-way repeated measures ANOVA, showed that there is a very similar efficacy in pain relief with both types of exercise according to the interventions of the two groups, as no statistically significant differences were found between interventions (F = 0.047; gl1 = 1; gl2 = 48; *p*-value = 0.829; ŋp^2^ = 0.001). These differences were not significant in any of the pairwise comparisons between groups in each evaluation (*p* > 0.387 for all evaluations). However, statistically significant differences were found over time (F = 165.762; gl1 = 1.821; gl2: 87.425; *p*-value < 0.001), with a very high effect size (ŋp^2^ = 0.775), decreasing pain by 58.17% in the GPR group and 63.16% in the STE group at the end of the study from the start of the intervention. When pairwise comparisons were performed, statistically significant differences were found, for both groups, between the pre-intervention assessments and the intermediate and final assessments, as well as between the intermediate and final assessments (*p* < 0.001 for all pairwise comparisons by post hoc Sidak test) (Figure 2).

### 3.2. Results of Pressure Pain Threshold (PPT)

The results of the evaluation of mechanosensitivity by measuring the pressure pain threshold indicated an improvement in pain intensity in both groups, with no differences between the interventions for all the evaluations performed (*p* > 0.341 for all the points of assessment). There was an increase in these thresholds, both locally in the MTrP assessment of the upper trapezius muscles and cervical vertebrae (C2 and C6) and remotely in the MTrP assessment of the anterior tibialis muscles. Considering pain thresholds to pressure, in all mechanosensitivity variables improvements were obtained with significant differences (*p* < 0.001), and with very high effect size (ŋp^2^ > 0.425 in all variables) (Table 3).

In the intra-group pairwise comparisons between the different assessments during the study, there was an almost linear increase in PPT in all variables during the 4 weeks of the study. Only no statistically significant differences were observed between the 3rd and 4th assessments in left C2 mechanosensitivity (*p* = 0.094).

### 3.3. Results of Craniocervical Angle (CCA)

The craniocervical angle, as an indicator variable of head position and postural control, has shown similar behavior to the rest of the pain variables. There was a slight increase in the degrees of CCA after the intervention in both groups. However, in the group with specific therapeutic exercise, significant differences did not appear until the last evaluation, at the end of the study (Table 4). In both groups the change at the end of the study was similar with a global high effect size (ŋp^2^ = 0.440), considering the differences in the evaluation moments (F = 37.738; gl1 = 2.288; gl2: 109.814; *p*-value < 0.001) and that there were no significant differences in the interaction group*evaluations (F = 0.723; gl1 = 3; gl2 = 46; *p*-value = 0.544).

### 3.4. Results of Pain-Related Psychosocial Factors

Data for the determination of Kinesiophobia and catastrophizing, as psychosocial factors associated with pain, were assessed at only three points in the study, at the two pre-intervention assessments and at the end of the interventions in both groups.

The behavior of both variables was the same, with a decrease in the values of kinesiophobia and catastrophizing very similar with both interventions, which again shows that there are no differences in the interaction time*group (kinesiophobia: F = 0.273; gl1 = 1.562; gl2 = 74.971; *p*-value = 0.707) (catastrophizing: F = 0.165; gl1 = 1.441; gl2 = 69.180; *p*-value = 0.776). However, significant differences and high effect size were detected between the evaluations (kinesiophobia: F = 8.928; gl1 = 1.562; gl2 = 74.971; *p*-value = 0.001; ŋp^2^ = 0.157) (catastrophizing: F = 19.115; gl1 = 1.441; gl2 = 69.180; *p*-value < 0.001; ŋp^2^ = 0.285), and no significant differences were obtained between interventions in kinesiophobia (F = 2.454; gl1 = 1; gl2 = 48; *p* = 0.124) and catastrophizing (F = 2.180; gl1 = 1; gl2 = 48; *p* = 0.146).

**Figure 3:**
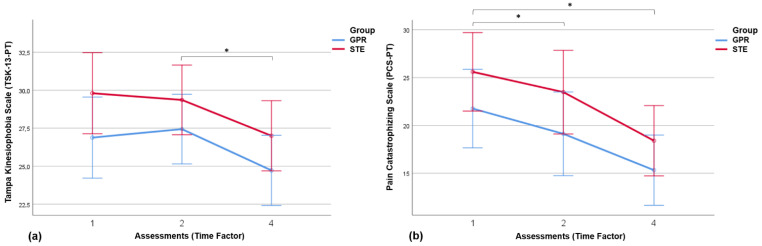
Longitudinal monitoring of kinesiophobia (**a**) and pain catastrophizing (**b**) in both intervention groups. GPR: Global Postural Re-education, STE: specific therapeutic exercise. * Statistically significant difference between assessments (pairwise comparisons by post hoc Sidak test (*p*-value < 0.05)).

Taking this into account, the decrease in kinesiophobia was close to 10% in both groups, and in intra-group pairwise comparisons by post hoc Sidak test, significant differences were obtained for two interventions between the second pre-intervention and the final assessment: in the GPR group (*p* = 0.013; CI: [0.474–4.966]) and in the STE group (*p* = 0.037; CI: [0.114–4.606]) (Figure 3a). The decrease in catastrophizing was 19.9% in the GPR group and 21.6% in the STE group. In pairwise comparisons, only within-group differences were found between the second pre-intervention and the final assessment (Sidak post hoc test) in the STE group (*p* = 0.026; CI: [0.491–9.669), while in the GPR group, within-group significant differences were obtained between the two pre-intervention assessments (*p* = 0.028; CI: [0.220–5.060]) and between the first pre-intervention and the final assessment (*p* = 0.002; CI: [2.068–10.812]) (Figure 3b).

## 4. Discussion

This study aimed to investigate the effectiveness of two different types of exercises, a global versus more specific exercise program in reducing pain, local and generalized mechanosensitivity, postural control with head position, and pain-related psychosocial factors in women with chronic nonspecific neck pain. Both interventions showed beneficial results in the treatment of chronic nonspecific neck pain, reducing pain perception, increasing mechanosensitivity, as well as improving attitudes, thoughts, and pain perceptions and their influence on movement by assessing catastrophizing and kinesiophobia.

The results indicate that both exercise modalities produced comparable benefits, suggesting that neither treatment was superior to the other. Consequently, both approaches can be considered viable short-term therapeutic options for managing nonspecific chronic neck pain.

Previous studies have highlighted the beneficial effects of GPR in individuals with neck pain, particularly when compared with a control group [110], static segmental stretching [111,112], or manual therapy [70,86]. However, the literature does not provide conclusive evidence regarding its effectiveness relative to other established exercise interventions. To date, no studies have directly compared GPR with alternative exercise-based treatments. The present study addresses this gap by contrasting GPR with a cervical-specific exercise program, which has already demonstrated efficacy in managing chronic neck pain [113,114,115]. The sample of this study has shown similar characteristics in both groups at the beginning of the study, which has allowed us to compare the efficacy of both interventions, with similar results.

### 4.1. Neck Pain

The change values considered as minimal clinically important difference (MCID) for mechanical neck pain for NPRS vary considerably according to different authors. For pain perception, measured with NPRS, MCID values range from 1.3 points in mixed neck pain [3] to 2.5 points in nonspecific neck pain [116]. The changes obtained in our study fall within these ranges with both types of exercises applied, with a decrease in pain measured with NPRS of 3.6 points in the GPR group and 3.8 points in the STE group. These findings indicate that both interventions resulted in clinically meaningful pain relief, suggesting that GPR and STE can be considered effective therapeutic options for CNSNP management. Given that both approaches led to similar reductions in pain, clinicians may select the intervention based on individual patient needs, accessibility, and professional expertise. But it is also important to consider, in addition to the absolute value of change, the size of the effect achieved and the percentage change with respect to baseline values. With the two interventions applied in our study, GPR and STE, a significant effect size has been obtained, with a very high effect size for the NPRS (ŋp^2^ = 0.775).

The data reported by Young et al. [117] on the change considered to be a clinically important difference (MCID) for mechanical neck pain are 1.5 points in NPRS. In our study, the decrease in NPRS values was higher both in absolute values (3.6 points in both interventions) and in percentage change (58.17% (GPR) and 63.17% (STE) compared with 56.86% reported by Young et al. [117]).

Beyond the observed reduction in pain intensity, the success of these interventions also depends on patient adherence and their feasibility in clinical settings. In this study, adherence was reinforced by supervision during treatment sessions and a structured home exercise program. However, in real-world conditions, factors such as patient motivation, perceived difficulty of exercises, and accessibility to therapy sessions could influence long-term adherence. Both interventions require professional supervision, but their integration into standard care may vary depending on healthcare resources and patient commitment. Future studies should explore strategies to enhance adherence and evaluate the practicality of incorporating these interventions into routine clinical practice to ensure long-term benefits.

### 4.2. Pressure Pain Threshold (PPT)

In addition to the NPRS as a subjective means of pain assessment, a semi-objective assessment of pain was performed using pressure algometry [118]. It is a reliable form of pain measurement for examining the sensitivity of different tissue levels, and the assessment of the short- and long-term effects of pain treatments on pain threshold to pressure [119]. Algometry is considered an evoked measure of static pain to assess the pain response to an applied pressure in a resting state [120,121,122]. The study of mechanosensitivity by assessing the pressure pain threshold has been used as an indicator in numerous studies on neck pain. Mainly applied locally to cervical muscles and vertebrae [51,123] and remotely in the tibialis anterior muscle [93,117] or first metacarpal joint [124] to evaluate widespread pain as a sign of central sensitization.

Our study showed that both exercise modalities used, GPR and STE, increased pressure pain thresholds at all points evaluated, both locally and remotely, and at all evaluations. These findings support the role of GPR and STE as effective interventions for modulating pain sensitivity, suggesting their potential impact on both peripheral and central sensitization mechanisms. Such improvements in mechanosensitivity are clinically relevant, as they may contribute to better pain management and functional outcomes in patients with CNSNP, reducing discomfort and enhancing tolerance to daily activities. The results obtained are in accordance with the literature, as well as with different interventions such as manual therapy [96,125,126,127], exercise [126,128,129], dry needling [130,131], or the combination of some of them such as manual therapy and exercise [124,132,133].

So far, beneficial results of GPR in pain reduction have been reported with subjective pain assessments, such as the visual analog scale or the NPRS [69,70,72,80], although no results have been shown on the effect of GPR on the pressure threshold to pain. With the results of our study, we can affirm that GPR has a short-term hypoalgesic effect on local (peripheral sensitization) and distance mechanosensitivity in CNSNP. This hypoalgesic effect reinforces the potential of GPR as a therapeutic approach to address pain sensitivity dysfunctions in patients with CNSNP. The observed increase in PPT may be attributed to GPR’s influence at both cortical and spinal levels, promoting greater cortical inhibition in peripheral muscles related to the affected segment [83]. Given its impact on central sensitization, further research should explore the long-term clinical implications of these findings.

### 4.3. Craniocervical Angle

The decrease in CCA is fundamentally related to a postural alteration, linked to increased mechanosensitivity due to reduced PPT [41,134].

In our study, the subjects treated with both interventions presented in the second pre-intervention evaluation a CCA of approximately 47 degrees, values similar to those shown by Martínez-Merino et al. [24], and in both groups there was a slight increase in the CCA at the end of the study: 51.56° in the GPR group and 49.80° in the STE group. This improvement in CCA suggests a shift toward a more neutral head posture, which is commonly targeted in postural correction interventions. Enhancing CCA can contribute to better spinal alignment, reducing excessive load on the cervical spine and promoting biomechanical efficiency. This increase in CCA corrects forward head posture and causes less mechanical stress on the tissues of the posterior suboccipital region [135], which in turn is related to an increase in PPT at high cervical levels [41].

Additionally, this postural correction may decrease the sustained mechanical strain on the C2 nerve root caused by suboccipital muscle shortening [136] and relieve stress on the articular surfaces of the upper cervical vertebrae [41,137].

These findings align with established postural correction protocols, which emphasize improving CCA to minimize mechanical overload on the cervical spine. A more neutral head position reduces compensatory stress on adjacent musculature and may enhance functional movement patterns, potentially lowering the risk of pain recurrence. Previous studies suggest that increasing CCA facilitates a more neutral head posture, reducing strain on posterior cervical structures and enhancing postural stability [26]. Both GPR and STE appear to follow these principles by inducing postural adjustments that may contribute to sustained functional improvements. The results of our study support the use of GPR as a therapeutic alternative for addressing mechanosensitivity related to postural alterations in CNSNP. This postural effect, along with improvements in postural control, has been previously documented, albeit with different outcome measures, in prior research [70,75,109].

### 4.4. Pain-Related Psychosocial Factors

Evidence has already shown that chronic pain is strongly related to kinesiophobia and induces a fear-avoidance behavioral change [15]. Similarly, pain catastrophizing, defined as an exaggerated negative cognitive and affective response to anticipated or actual pain, has been linked to altered pain modulation mechanisms and poorer functional outcomes [138]. Considering that kinesiophobia and pain catastrophizing can induce recurrence of neck pain or cause changes in the somatosensory system [139], it is important to consider them as indicators and outcome variables in the treatment of CNSNP, as suggested by some authors [140,141].

GPR has already shown in two previous studies an effect on kinesiophobia as a psychosocial factor in relation to pain, showing good results immediately after the intervention, although with a small increase in the score in a long-term effect (6 months). In our study, a slightly superior result in terms of effect size was obtained after treatment, being high, while in other studies, it was moderate [70,86].

The results with both types of exercise, GPR and STE, have been very similar, reducing both kinesiophobia and pain catastrophizing in the two intervention groups. The effect shown on kinesiophobia has been similar to that reported by Tejera et al. [142], obtaining in our study a high effect size (ŋp^2^ = 0.157) with a score decrease close to 10% in both groups. In the study by Tejera et al. [143], the effect was also very high, but the decrease in their values was greater than in our subjects, both in the virtual reality group (35%) and in the cervical-specific exercise group (18%) at one-month follow-up. Regarding pain catastrophizing, the decrease in the score in our study was close to 20% in both groups, whereas in the work of Tejera et al. [143], in both interventions the score decreased between 63.5% and 68.9%, with moderate-high effect size, as in our study.

In another study, which compared three different exercise modalities (neck-specific exercise, neck-specific exercise with behavioral intervention, and prescribed physical activity) [144], a moderate decrease in kinesiophobia, between 1 and 3 points, was obtained at a 6-month follow-up. In our study, after one month of intervention, kinesiophobia decreased between 2.4 points with STE and 2.7 points with GPR. We do not know the data of the subjects in the Peterson et al. study at the end of the first month, but with our results, we can suggest that GPR is a global therapeutic exercise modality at the level of other types of exercise.

In a multimodal intervention study in NSCNP, with three intervention groups and eight treatment sessions over 4 weeks [54], kinesiophobia scores decreased by 8 points when combining a protocol of manual therapy and therapeutic patient education, 5 points with a manual therapy protocol, and 1.6 points when combining manual therapy, therapeutic patient education, and a therapeutic exercise protocol. This suggests that, given our results, GPR may be a global exercise modality that could be used alone or in combination with manual therapy and/or therapeutic education for the treatment of NSCNP in patients with psychosocial factors associated with pain.

The clinical relevance of these findings lies in their potential long-term impact on patient functionality and treatment adherence [142]. Reducing kinesiophobia and pain catastrophizing may help patients break the cycle of fear-avoidance behaviors, allowing them to engage more confidently in physical activity and maintain functional independence [145]. This reduction in pain-related psychological distress can facilitate greater adherence to prescribed exercise programs, which has been associated with sustained symptom relief and a lower risk of relapse in chronic pain conditions. Additionally, improved self-efficacy and reduced fear of movement could promote long-term participation in physical activity, preventing disability progression and enhancing overall quality of life beyond the intervention period [146]. However, future research should focus on evaluating whether these psychological improvements translate into sustained functional benefits and on identifying the most effective strategies to support long-term adherence and optimize treatment outcomes.

The current evidence necessitates that further research is needed to study the effect of GPR, in addition to other exercise modalities, in isolation or in combination with other therapies, on different psychosocial factors, such as kinesiophobia and pain catastrophizing, as well as its choice depending on the presence of more or less psychosocial factors associated with NSCNP.

### 4.5. Limitations

This study presents several limitations. Firstly, the absence of an asymptomatic control group prevents direct comparisons between baseline measurements of the intervention groups and those of healthy individuals. Previous research suggests that participants exhibited mild to moderate pain and disability levels during pre-intervention assessments.

Another limitation concerns the inclusion of a home exercise program within both intervention protocols. Although participants were routinely questioned about their adherence during treatment sessions and supervised to ensure proper execution, no structured system was implemented to monitor the frequency and accuracy of home exercise performance.

Additionally, the study follow-up was limited to a short-term period of four weeks, restricting the ability to evaluate the long-term sustainability of the observed effects. Future research should aim to address these limitations by including a control group, implementing objective monitoring of home exercise compliance, and extending follow-up periods to assess the durability of treatment effects over the medium and long term. Furthermore, larger sample sizes would enhance the generalizability of the findings.

## 5. Conclusions

Global postural re-education, as a global postural exercise therapy modality, is as effective as a specific therapeutic exercise program in reducing subjective pain perception and reducing mechanosensitivity by increasing pressure pain thresholds locally and remotely in the short term. Therefore, GPR may be a treatment of choice for both peripheral and central sensitization in chronic nonspecific neck pain. GPR is also as effective as STE in reducing kinesiophobia and catastrophizing as psychosocial factors associated with chronic nonspecific neck pain. In addition, both interventions have a beneficial effect on postural control of the cervical spine by decreasing the craniocervical angle in the short term.

## Figures and Tables

**Table 1:** Baseline characteristics of the participants, demographic data, and outcome variables.

Variables	Intervention Groups (Mean ± Standard Deviation)			
	GPR Group (n = 25)	STE Group (n = 25)	Group Differences GPR-STE (*p*-Value)	
Age (years)	47.8 ± 8.9	53.8 ± 7.7	−5.9	(0.015) *
Weight (kg)	61.8 ± 6.9	62.9 ± 10.9	−1.1	(0.682)
Height (m)	1.61 ± 0.04	1.59 ± 0.06	0.01	(0.418)
BMI (kg/m^2^)	24 ± 3	24.6 ± 4.1	−0.75	(0.459)
NPRS	6.2 ± 1.4	6 ± 1.7	0.1	(0.783)
DNI	15.5 ± 5.4	16.1 ± 5.4	−0.6	(0.715)
CCA	47.4 ± 4.5	46.9 ± 7	0.5	(0.739)
TSK	26.9 ± 6	29.8 ± 7.2	−2.9	(0.126)
PCS	21.8 ± 10.1	25.6 ± 10.3	−3.8	(0.189)
PPT upper right Trapezius (kgf)	1.64 ± 0.75	1.75 ± 0.73	−0.11	(0.594)
PPT upper left Trapezius (kgf)	1.50 ± 0.60	1.44 ± 0.45	0.06	(0.650)
PPT right Tibialis Anterior (kgf)	2.66 ± 1.12	2.40 ± 0.88	0.26	(0.360)
PPT left Tibialis Anterior (kgf)	2.28 ± 0.93	2.34 ± 0.73	−0.06	(0.800)
PPT right C2 (kgf)	1.52 ± 0.43	1.56 ± 0.53	−0.04	(0.770)
PPT left C2 (kgf)	1.39 ± 0.40	1.64 ± 0.53	−0.25	(0.066)
PPT right C6 (kgf)	1.59 ± 0.45	1.73 ± 0.64	−0.14	(0.378)
PPT left C6 (kgf)	1.71 ± 0.68	1.74 ± 0.61	−0.03	(0.879)

GPR: Global Postural Re-education, STE: specific therapeutic exercise, BMI: body mass index, NPRS: Numerical Pain Rating Scale, CCA: craniocervical angle, TSK: Tampa scale kinesiophobia, PCS: pain catastrophizing scale, PPT: pressure pain threshold, C2: second cervical vertebrae, C6: sixth cervical vertebrae; * statistically significant difference between groups (independent *t*-test) (*p* < 0.05).

**Table 2:** Differences between two pre-intervention assessments of all outcome variables.

Variables	GPR Group 1st–2nd Assessments		STE Group 1st–2nd Assessments	
	Mean Difference (95% CI)	*p*-Value	Mean Difference (95% CI)	*p*-Value
NPRS	0.04 (−0.45, 0.53)	1.000	−0.04 (−0.53, 0.45)	1.000
DNI	1.56 (−0.08, 3.20)	0.067	0.72 (−0.92, 2.36)	0.632
CCA	0.32 (−0.86, 1.50)	0.975	0.24 (−0.94, 1.42)	0.994
TSK	−0.56 (−2.45,1.33)	0.848	0.44 (−1.45, 2.33)	0.918
PCS	2.64 (0.22, 5.06)	0.028 *	2.12 (−0.30, 4.54)	0.102
PPT Right Upper Trapezius	0.37 (0.09, 0.65)	0.004 †	0.31 (0.02, 0.60)	0.030 *
PPT Left Upper Trapezius	0.26 (0.01, 0.51)	0.041 *	0.15 (−0.12, 0.40)	0.540
PPT Right Tibialis Anterior	0.68 (0.25, 1.11)	0.000 †	0.22 (−0.21, 0.66)	0.650
PPT Left Tibialis Anterior	0.46 (0.13, 0.79)	0.003 †	0.26 (−0.07, 0.59)	0.207
PPT C2 (Right)	0.23 (0.05, 0.41)	0.006 †	0.19 (0.01, 0.37)	0.039 *
PPT C2 (Left)	0.15 (−0.01, 0.32)	0.081	0.26 (0.09, 0.42)	0.001 †
PPT C6 (Right)	0.22 (0.02, 0.42)	0.026 *	0.30 (0.10, 0.51)	0.001 †
PPT C6 (Left)	0.33 (0.09, 0.57)	0.003 †	0.33 (0.09,0.58)	0.003 †

GPR: Global Postural Re-education, STE: specific therapeutic exercise, NPRS: Numerical Pain Rating Scale, CCA: craniocervical angle, TSK: Tampa scale kinesiophobia, PCS: pain catastrophizing scale, PPT: pressure pain threshold, C2: second cervical vertebrae, C6: sixth cervical vertebrae, CI: confidence intervals. * Statistically significant difference (*p* < 0.05). † Statistically significant difference (*p* < 0.01).

**Table 3:** Within-group differences in the local and at-distance pressure pain thresholds from the second pre-intervention assessment.

ANOVA (Time Factor)		Intra-Group Pairwise Differences (Post Hoc Sidak Test)						
Location (PPT)	Within-Subject Effects	Group	2nd–3rd Assessments		2nd–4th Assessments		3rd–4th Assessments	
	(*p*-Value) ES (ŋp^2^)		Mean Difference (95% CI)	*p*-Value	Mean Difference (95% CI)	*p*-Value	Mean Difference (95% CI)	*p*-Value
Right Upper Trapezius	(0.000) (0.493)	GPR	−0.50 (−0.75, −0.26)	0.000 †	−1.08 (−1.42, −0.74)	0.000 †	−0.58 (−0.87, −0.29)	0.000 †
		STE	−0.37 (−0.62, −0.12)	0.001 †	−0.862 (−1.21, −0.51)	0.000 †	−0.49 (−0.79, −0.20)	0.000 †
Left Upper Trapezius	(0.000) (0.550)	GPR	−0.43 (−0.65, −0.212)	0.000 †	−0.90 (−1.20, −0.61)	0.000 †	−0.47 (−0.76, −0.19)	0.000 †
		STE	−0.35 (−0.58, −0.13)	0.001 †	−0.93 (−1.23, −0.63)	0.000 †	−0.58 (−0.87, −0.29)	0.000 †
Right Tibialis Anterior	(0.000) (0.425)	GPR	−0.70 (−1.11, −0.28)	0.000 †	−1.18 (−1.60, −0.77)	0.000 †	−0.49 (−0.86, −0.11)	0.005 †
		STE	−0.69 (−1.11, −0.27)	0.000 †	−1.20 (−1.62, −0.78)	0.000 †	−0.51 (−0.89, −0.14)	0.003 †
Left Tibialis Anterior	(0.000) (0.543)	GPR	−0.81 (−1.17, −0.45)	0.000 †	−1.44 (−1.87, −1.02)	0.000 †	−0.63 (−1.02, −0.24)	0.000 †
		STE	−0.60 (−0.96, −0.24)	0.000 †	−1.276 (−1.70, −0.86)	0.000 †	−0.68 (−1.07, −0.28)	0.000 †
C2 (Right)	(0.000) (0.559)	GPR	−0.42 (−0.62, −0.22)	0.000 †	−0.80 (−1.05, −0.56)	0.000 †	−0.38 (−0.61, −0.15)	0.000 †
		STE	−0.28 (−0.48, −0.08)	0.002 †	−0.70 (−0.95, −0.46)	0.000 †	−0.42 (−0.65, −0.20)	0.000 †
C2 (Left)	(0.000) (0.527)	GPR	−0.46 (−0.66, −0.27)	0.000 †	−0.74 (−0.98, −0.51)	0.000 †	−0.28 (−0.52, −0.04)	0.016 *
		STE	−0.36 (−0.56, −0.17)	0.000 †	−0.58 (−0.82, −0.32)	0.000 †	−0.22 (−0.46, −0.02)	0.094
C6 (Right)	(0.000) (0.541)	GPR	−0.37 (−0.61, −0.13)	0.001 †	−0.96 (−1.28, −0.65)	0.000 †	−0.59 (−0.88, −0.30)	0.000 †
		STE	−0.42 (−0.66, −0.18)	0.000 †	−0.85 (−1.17, −0.53)	0.000 †	−0.43 (−0.72, −0.14)	0.001 †
C6 (Left)	(0.000) (0.492)	GPR	−0.34 (−0.60, −0.09)	0.003 †	−0.78 (−1.02, −0.532)	0.000 †	−0.43 (−0.67, −0.20)	0.000 †
		STE	−0.41 (−0.66, −0.16)	0.000 †	−0.88 (−1.12, −0.63)	0.000 †	−0.46 (−0.70, −0.23)	0.000 †

PPT: pressure pain threshold, ES: effect size, ŋp^2^: partial eta squared, CI: confidence intervals, GPR: Global Postural Re-education, STE: Specific Therapeutic Exercise. C2: second vertebra, C6: sixth vertebra. * Statistically significant difference (*p* < 0.05). † Statistically significant difference (*p* < 0.01).

**Table 4:** Within-group differences of the craniocervical angle from the second pre-intervention assessment.

ANOVA (Time Factor)		Intra-Group Pairwise Differences (Post Hoc Sidak Test)						
Variable	Within-Subject Effects	Group	2nd–3rd Assessments		2nd–4th Assessments		3rd–4th Assessments	
	(*p*-Value) ES (ŋp^2^)		Mean Difference (95% CI)	*p*-Value	Mean Difference (95% CI)	*p*-Value	Mean Difference (95% CI)	*p*-Value
CCA	(0.000) (0.440)	GPR	−1.5 (−2.7, −0.3)	0.008 *	−4.4 (−6.2, −2.7)	0.000 †	−3 (−4.6, −1.3)	0.000 †
		STE	−0.8 (−2, 0.3)	0.299	−3.2 (−4.9, −1.4)	0.000 †	−2.3 (−4, −0.7)	0.002 *

ES: Effect size, ŋp^2^: partial eta squared, CI: confidence intervals, CCA: craniocervical angle, GPR: Global Postural Re-education, STE: specific therapeutic exercise. * Statistically significant difference (*p* < 0.05). † Statistically significant difference (*p* < 0.01).

## Data Availability

Data are contained within the article.

## Trial Registration

ClinicalTrials.gov ID: NCT04402463. Prospectively registered (data 22 May 2020).
